# The Impact of Cerebral Small Vessel Disease on Functional Recovery After Intracerebral Hemorrhage: Stratified Analysis by Age

**DOI:** 10.3390/jcm14186450

**Published:** 2025-09-12

**Authors:** Hong-Jae Lee, Haney Kim, Sook Joung Lee

**Affiliations:** 1 Department of Neurosurgery, Daejeon St. Mary’s Hospital, College of Medicine, The Catholic University of Korea, Seoul 06591, Republic of Korea; kosailee73@catholic.ac.kr; 2 College of Medicine, The Catholic University of Korea, Seoul 06591, Republic of Korea; harneykim@gmail.com; 3 Department of Physical Medicine and Rehabilitation, Daejeon St. Mary’s Hospital, College of Medicine, The Catholic University of Korea, Seoul 06591, Republic of Korea

**Keywords:** small vessel disease, intracerebral hemorrhage, rehabilitation, functional outcome

## Abstract

**Background**: Cerebral small vessel disease (cSVD) is a major contributor to intracerebral hemorrhage (ICH). Its presence carries significant implications for stroke prevention, acute management, post-stroke recovery, and socioeconomic burden. Despite its clinical significance, the impact of cSVD on functional outcomes after ICH, particularly concerning aging, remains uncertain. **Objective**: This study evaluated how cSVD influences post-ICH functional recovery, using age stratification (<65 and ≥65 years) and a multidomain functional assessment approach. **Methods**: We retrospectively analyzed data from 356 patients with primary spontaneous ICH. Functional status was evaluated at baseline and at three months post-ICH across multiple domains, including global disability, activities of daily living, gait, upper-extremity function, and swallowing ability, using validated assessment tools. Patients were categorized based on age and the presence or absence of cSVD. **Results**: Patients without cSVD consistently exhibited better functional status than those with cSVD at both baseline and three-month evaluations across age groups. Although all groups showed statistically significant functional improvement over time, the degree of improvement was significantly lower in patients with cSVD, particularly among those aged 65 years or older. Multivariable logistic regression analysis confirmed that cSVD was a strong and independent predictor of poor functional outcomes at three months after ICH. **Conclusions**: Our findings emphasize that cSVD is not merely a passive comorbidity but an active and independent determinant of poor prognosis and limited recovery following ICH. The clinical importance of early detection of cSVD supports the need for more intensive, individualized rehabilitation strategies in ICH survivors.

## 1. Introduction

Cerebral small vessel disease (cSVD) is increasingly recognized as a major contributor to ischemic stroke, spontaneous intracerebral hemorrhage (ICH), and neurodegenerative disorders in older adults [1]. In most ICH cases, particularly in the elderly and in spontaneous, non-traumatic primary ICH related to cSVD, cSVD presents a catastrophic form of stroke associated with high mortality, significant morbidity, and high risk of recurrence [2,3].

cSVD is associated with vascular dementia, gait disturbance, behavioral disorders, and functional disabilities [1], and it also shares conventional vascular risk factors including age, hypertension, diabetes mellitus, and smoking [4,5]. Despite its high prevalence and clinical significance, cSVD is often considered a “clinically silent” condition until it manifests through severe cerebrovascular events such as ischemic or hemorrhagic stroke [2]. However, the presence of cSVD has enormous implications for stroke prevention, acute management, post-stroke recovery, recurrence, and overall socioeconomic burden. Previous studies have revealed that ICH is caused by cSVD in a 85% of cases [4,6] and can also lead to poor prognosis [7]. Notably, cSVD has profound implications across all stages of ICH.

Pathologically, cSVD encompasses all conditions affecting the cerebral microcirculatory vasculature, including the small arteries, arterioles, capillaries, venules, and small veins [8]. It primarily involves the deep white and gray matter of the brain [9,10], where perforating vessels play a critical role in supporting the brain’s most metabolically active nuclei and complex neural networks [11]. Importantly, “small vessel disease” (SVD) is not limited to the brain; it can also involve any microvascular structures in the human body, such as the retina, heart, lung, and kidneys, reflecting a systemic vascular condition [12]. Thus, the presence of SVD in any organ implies that the patient is already suffering from a “systemic” condition [12]; therefore, cSVD implies a systemic disease.

Throughout such cases, although “clinically silent” until hemorrhagic stroke has developed, the presence of cSVD has profound implications across all the stages of ICH. Furthermore, as a manifestation of “systemic” small vessel pathology, cSVD reflects a broader vascular vulnerability that may influence stroke outcomes beyond the acute phase.

While the association between cSVD and ICH has been well-documented in prior studies [13,14], most research has focused on neuroimaging findings, as well as the risk of hematoma expansion and recurrence [4,7,15,16]. Unfortunately, functional assessment in previous studies was largely limited to the modified Rankin scale (mRS) [13,17]. As is well recognized, functional recovery encompasses a broad spectrum, including gait, swallowing, cognition, and activities of daily living. Despite its clinical importance, the specific impact of cSVD on functional recovery after ICH remains poorly understood. In particular, the interaction between cSVD and aging, which may critically shape recovery trajectories, has not been thoroughly investigated.

This study aimed to investigate the impact of cSVD on functional improvement after ICH through a comprehensive assessment of multiple functional domains. Since both ICH and cSVD are significantly influenced by age [1,3,8], patients were stratified into two subgroups (<65 and ≥65 years) to minimize confounding effects.

## 2. Materials and Methods

This study was designed as a retrospective chart review. Consecutive patients with spontaneous ICH admitted to our medical university hospital between 1 January 2020 and 31 December 2024 were screened for this study. Patients with traumatic ICH or hemorrhagic transformation of cerebral infarction were excluded. Patients with a history of previous stroke that could affect functional decline were also excluded. In addition, patients with unavailable or unreliable CT scans, bilateral or multiple ICH sites (due to ICH classification), and those who died within 7 days after ICH onset were excluded.

Patients underwent MRI studies to rule out secondary causes of ICH, including arteriovenous malformations (AVMs), tumor, and other structural abnormalities. After initial management by the neurosurgical team—either surgical or conservative—rehabilitation therapy was initiated when patients became neurologically stable, typically within 2 weeks of ICH onset. Individualized rehabilitation included physical and occupational therapy, with additional dysphagia, cognitive, or speech therapy as needed.

### 2.1. Neuroimaging Classification of ICH and cSVD

CSVD was identified based on characteristic neuroimaging findings on CT or MRI, including lacunes and white matter changes, as assessed by an experienced neuroradiologist. The diagnosis incorporated both clinical and imaging data. cSVD subtypes were classified according to the STRIVE criteria and previous studies [2], including recent small subcortical infarct, lacune, white matter hyperintensity, perivascular spaces, cerebral microbleeds, and brain atrophy.

ICH etiology was classified based on neuroimaging and clinical data into the following categories: hypertension-related ICH, cerebral amyloid angiopathy (CAA), arteriovenous malformation (AVM), moyamoya disease (MMD), tumor-related hemorrhage, and ICH of unknown cause [18]. ICH location was categorized as basal ganglia, thalamus, cerebral lobe, pons and brainstem, or cerebellum, following previous studies [18,19].

Neuroimaging was assessed to classify ICH etiology and location, as well as to confirm the presence of SVD. Subsequently, ICH and SVD scores were calculated using standardized criteria. ICH score [20] is a validated prognostic grading scale (0–6 points) that incorporates GCS, hemorrhage volume, intraventricular extension, infratentorial origin, and age ≥ 80 years to predict 30-day mortality in spontaneous ICH patients. The SVD score (0–4) [21] was assigned by summing the presence of lacunes, microbleeds, white matter hyperintensities (WMHs), and enlarged perivascular spaces on MRI. Patients without MRI were excluded from SVD score analysis.

### 2.2. Functional Evaluation

Functional evaluations were conducted at the start of rehabilitation (within 2 weeks of ICH onset) and at 3 months after rehabilitation. At our medical center, comprehensive functional assessment across multiple functional domains is part of the routing rehabilitation protocol, thus all patients undergo standardized evaluations accordingly. The assessments included global disability (modified Rankin scale, mRS), activities of daily living (modified Barthel index, MBI), balance and gait (Berg balance scale, BBS), gait function (functional ambulatory category, FAC), upper-extremity function (manual function test, MFT), and swallowing function.

The mRS is a 7-point scale measuring global disability after stroke, from 0 (no symptoms) to 6 (death) [22] with scores of 0–2 indicating good outcomes. The modified Barthel index (MBI) is a widely used scale for assessing independence in activities of daily living (ADL), such as feeding, bathing, bowel and bladder control, gait, and stair climbing, with higher scores indicating greater independence [23]. The BBS is a 14-item objective measure that assesses static and dynamic balance abilities, with higher scores reflecting better balance function [24]. The FAC categorizes walking ability into six levels based on required assistance [25], while the MFT evaluates upper-limb motor function through tasks such as grasping, pinching, and object manipulation, commonly used in stroke [26]. Higher FAC and MFT scores also indicate better function. Swallowing function was assessed using the videofluoroscopic swallowing study (VFSS), the gold standard for evaluating dysphagia.

### 2.3. Statistical Analysis

Statistical analyses were performed using IBM SPSS Statistics, version 29.0 (IBM Corp., Armonk, NY, USA). Patients were stratified by age and cSVD status. Within each subgroup, categorical variables were compared between patients with and without SVD using the Chi square test or Fisher’s exact test, as appropriate, while continuous variables were compared using Student’s *t*-test. Multivariable logistic regression analyses were performed using different dependent variables (e.g., poor prognosis or SVD status) to explore various associations with potential predictors.

The study protocol was approved by the Institutional Review Board (IRB) and the Ethics Committee of our hospital (DC25RISI0033), and informed consent was waived due to the retrospective study design and minimal harm to the patients.

## 3. Results

**Figure 1:**
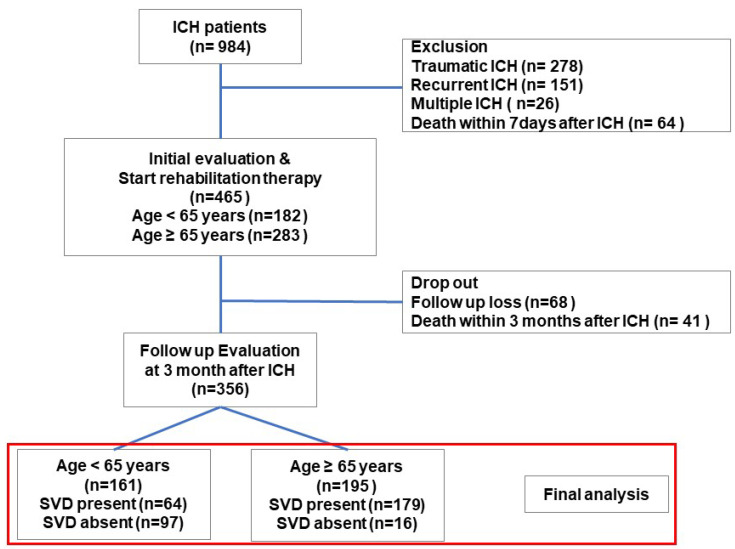
Flowchart of patient selection.

A total of 984 patients were admitted with ICH between 2020 and 2024. After excluding cases of traumatic, recurrent, or multiple ICH, and early mortality, 465 patients with spontaneous ICH were identified. Of these, 356 patients who underwent both initial and three-month post-ICH functional evaluations were included in the final analysis. This cohort study consisted of 161 patients aged under 65 years and 195 patients aged 65 years or older (Figure 1).

A total of 984 patients were admitted with intracerebral hemorrhage (ICH). After excluding patients with traumatic, recurrent, or multiple ICH and those with early mortality, 465 cases of spontaneous ICH were identified. Among these, 356 patients who completed both initial and three-month functional evaluations were included in the final analysis.

Table 1 presents the demographic and baseline clinical characteristics of patients with ICH stratified by age group and presence of cSVD. Among patients aged under 65 years, those with cSVD were significantly older and were more likely to be male and had higher rates of hypertension and diabetes mellitus compared to those without cSVD. The prevalence of chronic kidney disease and smoking was also significantly higher in the cSVD group (all *p* < 0.05).

In patients aged 65 years or older, those with cSVD were significantly older and had a higher prevalence of hypertension and chronic kidney disease compared to those without cSVD. Across all age groups, the presence of cSVD was significantly associated with higher ICH scores and poorer functional outcomes at three months after ICH (all *p* < 0.05).

Neuroimaging characteristics revealed that hypertension-related ICH was more prevalent among younger patients with cSVD, whereas CAA was significantly more common in older patients with cSVD (Table 2). In addition, Moyamoya disease was significantly more prevalent in patients with cSVD across both age groups. In terms of ICH location, thalamic involvement was significantly more common among younger patients with cSVD. Notably, 82.57% of ICH cases in this group were located in the subcortical or deep brain regions.

Regarding neuroimaging markers of cSVD, WMHs and cerebral microbleeds were the most frequently observed in patients under 65 years (76.6% and 51.6%, respectively), whereas WMHs and lacunes were predominant in those aged 65 years or older (84.9% and 50.8%, respectively). The total SVD score was higher in the older cSVD group compared to the younger cSVD group (2.18 ± 0.90 vs. 1.73 ± 0.67).

Table 3 summarizes the between-group comparisons of functional evaluations at the initial and three-month follow-up, stratified by age and cSVD status. Among patients aged under 65 years, those with cSVD had significantly higher initial mRS scores and lower initial MBI, BBS, and MFT scores compared to those without cSVD. At three months after ICH, patients with cSVD continued to demonstrate poorer functional outcomes, with significantly higher mRS scores and lower MBI and hand function scores (all *p* < 0.05). Swallowing function also showed a significant difference at three months, while 91.8% of patients without cSVD resumed a normal diet, only 70.3% of those with cSVD did so, with a higher proportion remaining on limited or non-oral diets (*p* = 0.002).

Similarly, in patients aged 65 years or older, the cSVD group had significantly higher initial mRS scores and lower MBI and BBS scores. At three months after ICH, functional recovery in the cSVD group remained significantly poorer across all domains, with higher mRS scores and lower MBI, BBS, FAC, and MFT scores (all *p* < 0.05). Swallowing function was also notably impaired in this older group: at three months, 54.8% of patients with cSVD remained on non-oral feeding, whereas none of the patients without cSVD remained on non-oral feeding (*p* = 0.021). Only 17.3% of those with cSVD were able to resume a normal diet, indicating that cSVD was associated with markedly delayed recovery of swallowing function.

**Figure 2:**
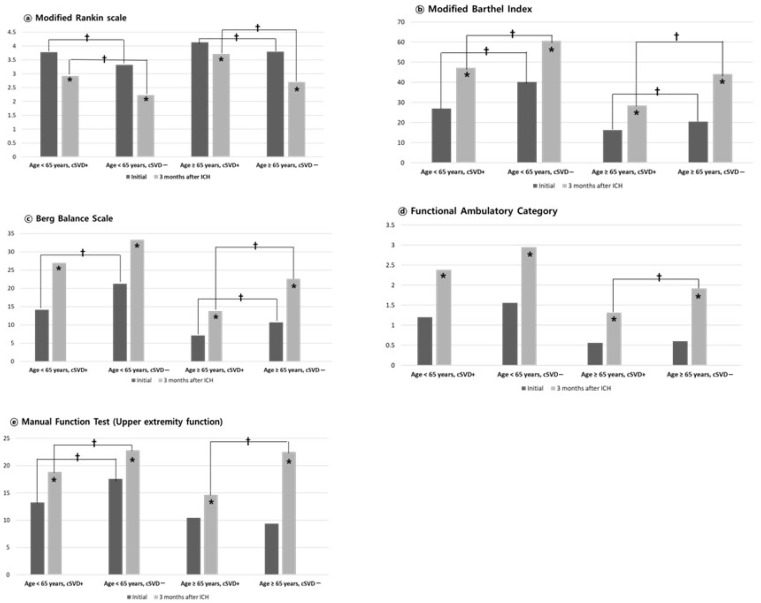
**Functional improvement in each domain stratified by age and cSVD status**. Functional evaluations for each domain ((**a**): modified Rankin scale (mRS), (**b**): modified Barthel index (MBI), (**c**): Berg balance scale (BBS), (**d**): functional ambulation category (FAC), (**e**): manual function test (MFT)) are presented as bar charts showing mean values at initial assessment and at three months after ICH. Data are stratified by age group (<65 years, ≥65 years) and presence or absence of cSVD. Within-group analyses demonstrated significant functional improvement from baseline to three months in all groups (indicated as “*”, * *p* < 0.05, paired *t* test). Between-group comparisons revealed that, at both initial and three-month follow-up, functional status was consistently better in patients without cSVD across both age group strata (indicated as “†”, † *p* < 0.05, Student’s *t*-test). * *p* < 0.05 indicated significant within-group improvement between initial and three-month evaluations (paired *t*-test), while † *p* < 0.05 indicated significant between-group difference at initial and three-month evaluations (Student’s *t*-test).

Figure 2 illustrates functional evaluations for each domain at initial assessment, and three-month follow-up, according to age group (<65 years and ≥65 years) and cSVD status. In the within-group analysis, all groups—including those aged under and over 65 years, and both cSVD and non-cSVD groups—achieved significant functional improvement between initial and three-month evaluations across all functional domains (all *p* < 0.05; indicated as “*” in Figure 2). As also shown in Table 3, functional status at both initial and three-month evaluations was consistently and significantly better in the non-cSVD group compared to the cSVD group across both age groups.

**Figure 3:**
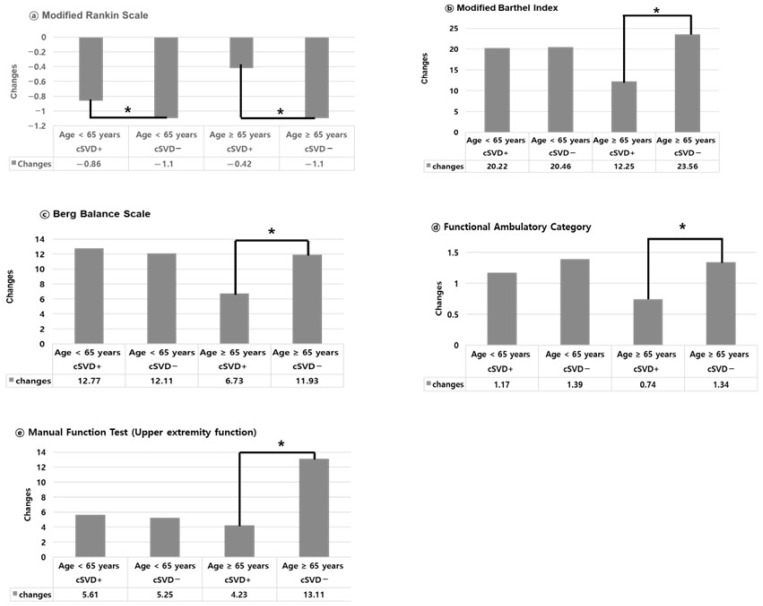
**Degree of functional improvement (“changes”) stratified by age and cSVD status.** Bar charts display the mean changes in functional scores between baseline and three months after ICH across five domains: (**a**) modified Rankin scale (mRS), (**b**) modified Barthel index (MBI), (**c**) Berg balance scale (BBS), (**d**) functional ambulation category (FAC), and (**e**) manual function test (MFT, upper-extremity function). Data are stratified by age (<65 years vs. ≥65 years) and presence or absence of cSVD. Significant between-group differences in functional improvement were observed only in the elderly group (≥65 years), where patients without cSVD achieved significantly greater improvement across all functional domains (indicated as “*” * *p* < 0.05, Student’s *t* test). In patients aged <65 years, the degree of improvement did not significantly differ between cSVD and non-cSVD groups, except for mRS. *: *p* < 0.05, significant between-group difference in change values (Student’s *t*-test).

To assess the extent of improvement within each group, we defined “*change*” as the difference between the three-month score and the initial score (three-month value—initial value) for each functional domain. When comparing the degree of functional improvement “changes” in Figure 3, significant between-group differences were observed only in patients aged ≥65 years. Specifically, elderly patients without cSVD demonstrated significantly greater improvements across all functional domains (all *p* < 0.05).

In contrast, among patients aged under 65 years, the degree of improvement was similar between groups regardless of cSVD status, and no significant differences were observed in any functional domains except for the mRS (Figure 3).

Table 4 demonstrates the results of multivariable logistic regression analysis for predictors of poor prognosis (defined as mRS 3–5 at three months after ICH), stratified by age group. In patients aged <65 years, the presence of cSVD was significantly associated with poor prognosis (OR = 3.82, 95% CI: 1.23–8.76, *p* = 0.004), as was a higher ICH score (OR = 3.95, 95% CI: 2.51–6.21, *p* < 0.001). Additionally, the presence of CKD (OR = 4.21, 95% CI: 1.35–7.61, *p* = 0.002), hypertension (OR = 2.95, 95% CI: 1.05–4.26, *p* = 0.03), and diabetes (OR = 3.16, 95% CI: 1.12–6.94, *p* = 0.02) was also significantly associated with poor outcomes. In contrast, age and heart failure were not significant predictors in this age group.

In patients aged ≥65 years, cSVD remained a strong independent predictor of poor prognosis (OR = 7.44, 95% CI: 2.40–15.35, *p* < 0.001), along with a higher ICH score (OR = 3.29, 95% CI: 2.36–4.89, *p* < 0.001). The presence of CKD (OR = 5.48, 95% CI: 1.43–13.72, *p* = 0.030) and hypertension (OR = 2.17, 95% CI: 1.74–3.49, *p* < 0.001) were also significantly associated with poor outcomes. However, age, diabetes, and heart failure were not significantly associated with prognosis in this older population.

## 4. Discussion

In this study, we investigated the impact of cSVD on functional recovery after ICH by comprehensively evaluating multiple functional domains, including global disability (mRS), activities of daily living, gait, upper-extremity function, and swallowing. To minimize the confounding effect of age, patients were stratified into two subgroups: those aged <65 years and those aged ≥65 years.

In our large cohort of primary spontaneous ICH patients, those without cSVD consistently exhibited better functional status than those with cSVD at both initial and three-month evaluations across both age groups. Notably, although all groups—regardless of age or cSVD status—achieved statistically significant functional improvement over time, the degree of improvement was significantly lower in patients with cSVD, particularly among those aged 65 years or older. These findings suggest that cSVD has a more pronounced negative impact on post-stroke recovery in older patients.

Multivariable logistic regression analysis (Table 4) further confirmed that the presence of cSVD was a strong and significant independent predictor of poor functional outcomes at three months after ICH even after adjusting for other variables such as ICH score, age, and comorbidities. This association was stronger in the older population, indicating that cSVD may play a critical role in determining long-term outcomes in elderly ICH survivors.

### 4.1. Distinct Contributions Compared to Previous Studies

Previous studies have identified cSVD as a key etiological factor for ICH, particularly in elderly patients, and is associated with poor clinical outcomes [5,13,15,27,28]. Uniken et al. [13] reported that cSVD significantly worsened functional outcomes after ICH, even after adjusting for hematoma volume and location. Similarly, Cheng et al. [14] found that cSVD markers—such as white matter hyperintensities and lacunes—were significantly associated with reduced functional recovery. Sakamoto et al. [29] also noted that higher cSVD burden and older age were independently associated with deep ICH locations, suggesting a shared microangiopathic mechanisms.

Our findings align with prior studies demonstrating the detrimental impact of cSVD on ICH while offering several distinct contributions. First, we stratified patients by age, revealing that the detrimental impact of cSVD is evident even in younger adults, though more pronounced in the elderly. Second, unlike many studies that relied solely on the mRS as a functional outcome measure [7,13,14,15,29,30], our study comprehensively assessed multiple functional domains—including daily activities, balance and gait, upper-extremity function, and swallowing—using validated tools such as the MBI, BBS, FAC, MFT, and VFSS. This multidimensional approach offers a more comprehensive understanding of the functional burden of cSVD following ICH.

Our methodological strengths highlight the independent role of cSVD in limiting recovery after ICH and support the need for age-specific rehabilitation strategies.

### 4.2. Age-Dependent Impact of cSVD on Functional Recovery After ICH

Notably, our findings highlight the age-dependent influence of cSVD on functional recovery following ICH. While cSVD was associated with poorer functional status at both baseline and three-month evaluations across all age groups, its impact was particularly pronounced in patients aged ≥65 years. Among younger patients, cSVD had a limited effect on the degree of functional improvement (Figure 2 and Figure 3). In contrast, older patients with cSVD exhibited significantly attenuated recovery across multiple functional domains indicating that cSVD imposes a broader and more detrimental effect in this age group.

This age-specific vulnerability may reflect age-related reductions in neuroplasticity and brain resilience, which amplify the adverse effects of cSVD pathology.

Previous studies have established cSVD as a major contributor to spontaneous ICH in older adults and a strong predictor of cognitive and functional decline [1,4,6]. It also shares conventional vascular risk factors, including age, hypertension, diabetes, and smoking [4,5]. cSVD becomes increasingly prevalent with age, affecting most individuals over 60 and nearly all those over 90 [31]; however, the interaction between age and the functional impact of cSVD has not been clearly delineated. Our study directly addressed this gap by stratifying patients by age and employing multidomain functional assessments. Multivariable logistic regression confirmed that cSVD was a strong and independent predictor of poor outcomes in older ICH survivors.

The negative impact of cSVD on recovery is amplified in the elderly, likely due to the cumulative burden of microvascular injury and concurrent age-related neurodegenerative processes [12,25]. Pathophysiological mechanisms implicated in aging-related cSVD include endothelial dysfunction, blood–brain barrier breakdown, chronic low-grade inflammation (“inflammaging”), and reduced cerebral perfusion—all of which contribute to impaired neuroplasticity and delayed recovery [28,32,33].

Clinically, our results underscore the importance of early recognition of cSVD in older patients with ICH. Identifying cSVD at admission may improve prognostic accuracy, guide realistic goal setting, and support individualized rehabilitation strategies. Elderly patients with cSVD may particularly benefit from more intensive rehabilitation, closer monitoring, and early counseling of patients and families regarding expected outcomes.

### 4.3. Considerations for the Elderly Without cSVD Subgroup

In our cohort, only 16 elderly patients (≥65 years) without cSVD were identified. Although this small number inevitably limits the robustness of subgroup analyses, this finding is consistent with prior population-based studies reporting a high prevalence of cSVD markers in older adults, with up to 80% of individuals aged ≥60 years demonstrating white matter hyperintensities [34], and nearly 70% of those aged 65–74 years exhibiting at least one cSVD marker [35]. From a clinical perspective, it is also uncommon to encounter elderly patients without any radiological evidence of cSVD—including WMHs, lacunes, brain atrophy, or enlarged perivascular spaces. Therefore, while the elderly non-cSVD subgroup in our study was small, this distribution likely reflects the true clinical reality rather than a sampling artifact. These considerations should be borne in mind, and the results interpreted with appropriate caution.

### 4.4. Functional Impact of cSVD in Younger Adults

While the detrimental effect of cSVD on functional recovery has been predominantly observed in older adults, our study demonstrates that cSVD also adversely affects recovery in younger patients (<65 years). Despite generally higher recovery potential in younger ICH individuals [36], those with cSVD exhibited significantly poorer functional status at both the initial and three-month evaluations compared to those without cSVD (Table 3, Figure 2 and Figure 3). Although between-group differences in the degree of functional improvement were less marked in the younger cohort, the persistently lower scores in the cSVD group indicate a sustained functional disadvantage attributable to microvascular pathology even in younger brains. These findings are consistent with prior studies [37,38], which reported that cSVD is not uncommon in young adults with ICH and may impair recovery regardless of age. Consistent with previous studies [37,38], our study found that cerebral microbleeds and white matter changes were the most frequently observed cSVD markers in younger ICH patients. This underscores the clinical relevance of detecting and monitoring cSVD in younger ICH patients, as early recognition could inform risk stratification and personalized rehabilitation planning, even in populations traditionally considered to have favorable prognoses.

As shown in Table 1, younger patients with cSVD had significantly higher rates of conventional vascular risk factors such as hypertension, diabetes mellitus, chronic kidney disease, and smoking history compared to those without cSVD. As previously mentioned, SVD is being increasingly recognized as a “systemic disease”, and cSVD shares many of the same vascular risk factors. These findings suggest that even in younger patients, the presence of cSVD may reflect a broader systemic vascular burden. Accordingly, early identification and aggressive management of underlying comorbidities may be essential to improving outcomes in this population.

Additionally, baseline differences in comorbidity burden were observed between younger patients with and without cSVD, which may have introduced potential confounding effects. To address this concern, we performed sensitivity analyses adjusting for these baseline factors, and the results remained consistent with our primary findings. This indicates that the adverse impact of cSVD on functional outcomes in younger adults cannot be fully explained by comorbidities alone, highlighting cSVD itself as an independent determinant of recovery.

### 4.5. Study Limitations

This study has several limitations. First, its retrospective and single-center design may introduce selection bias and limit generalizability. In particular, because only patients who completed both the initial and three-month evaluations, those with the worst outcomes, including patients who died within seven days after ICH (*n* = 64), those who died within three months (*n* = 41), and those lost to follow-up (*n* = 68)—were excluded. This selection process may have introduced survival bias and potentially biased our cohort toward survivors with relatively better outcomes. Second, the relatively small number of patients in certain subgroups—particularly elderly individuals without cSVD—may have reduced statistical power for subgroup analyses. Third, baseline differences in comorbidity burden between groups may have introduced potential confounding effects. Fourth, because multiple functional domains were assessed, the risk of type I errors due to multiple comparisons cannot be excluded. Finally, cognitive function and aphasia were not assessed due to limited data availability and evaluation constraints in retrospective settings. In addition, cognitive impairment and aphasia were not consistently distinguished in clinical records, which limited the reliability of data extraction during chart review.

Although this study has several limitations, it also has notable strengths, including consistent patient management and comprehensive multidomain functional evaluations performed under standardized protocols in a single tertiary center, which enhanced internal validity. Importantly, further large-scale, multicenter prospective validation studies are warranted to confirm these findings, and to better clarify the causal relationship between cSVD and functional recovery after ICH. In addition, these studies should incorporate standardized cognitive and language (aphasia) assessments to provide a more comprehensive evaluation of outcomes.

## 5. Conclusions

This study investigated the impact of cSVD on functional recovery after ICH through a multidimensional assessment of functional domains and an age-stratified analysis. Our findings demonstrated that cSVD significantly influenced both the initial neurological severity and the degree of functional improvement after ICH—particularly in elderly patients. These results suggest that cSVD is not merely a passive comorbidity, but an active and independent determinant of poor prognosis and limited recovery. Given that cSVD often remains clinically silent prior to stroke onset, routine screening—especially in the elderly—may facilitate risk stratification and inform post-ICH management strategies. Incorporating cSVD status into prognostic models may further improve the precision of outcome prediction and support long-term care planning in this population. Overall, our findings highlight the clinical importance of the early detection of cSVD and support the need for more intensive, individualized rehabilitation approaches in ICH survivors. We hope that our findings may help inform future research directions exploring the role of cSVD in ICH recovery and outcomes.

## Figures and Tables

**Table 1:** Demographic factors and clinical characteristics of patients with ICH.

	Age < 65 Years (*n* = 161)			Age ≥ 65 Years (*n* = 195)		
	cSVD+ (*n* = 64)	cSVD− (*n* = 97)	*p*-Value	cSVD+ (*n* = 179)	cSVD− (*n* = 16)	*p*-Value
Age (year)	54.67 ± 8.44	48.68 ± 10.82	<0.001	78.90 ± 7.59	67.1 ± 3.9	<0.001
Male, *n* (%)	45 (70.31)	56 (57.73)	0.03	104 (58.10)	7 (43.75)	0.051
Hypertension, *n* (%)	57 (89.06)	61 (62.89)	0.022	173 (96.65)	8 (50.0)	<0.001
Diabetes, *n* (%)	31 (48.44)	16 (16.49)	<0.001	105 (58.66)	4 (25.0)	0.342
Hyperlipidemia, *n* (%)	32 (50.0)	27 (27.84)	0.077	114 (63.69)	5 (31.25)	0.353
Atrial fibrillation, *n* (%)	3 (4.69)	2 (2.06)	0.744	35 (19.55)	1 (6.25)	0.564
Heart failure, *n* (%)	6 (9.38)	4 (4.12)	0.541	43 (24.02)	0 (0.0)	0.569
Chronic kidney disease, *n* (%)	4 (6.25)	0 (0.0)	0.020	21 (11.73)	2 (12.5)	<0.001
Antiplatelet use, *n* (%)	11 (17.19)	7 (7.22)	0.807	89 (49.72)	4 (25.0)	0.049
Anticoagulant use, *n* (%)	7 (10.94)	4 (4.12)	0.122	27 (15.08)	2 (12.5)	0.553
Alcohol use, *n* (%)	51 (79.69)	59 (60.82)	0.067	61 (34.08)	5 (31.25)	0.014
Smoker, *n* (%)	35 (54.69)	43 (44.33)	0.037	41 (22.91)	4 (25.0)	0.006
History of cognitive impairment, *n* (%)	4 (6.25)	0 (0.0)	<0.001	62 (34.64)	2 (12.5)	<0.001
ICH score (0–4)	1.84 ± 1.50	1.54 ± 1.38	0.041	2.20 ± 1.31	1.5 ± 1.35	0.007
SVD score (0–6)	1.73 ± 0.67	NA	NA	2.18 ± 0.90	NA	NA
Good prognosis (mRS 0–2) 3 month after ICH, *n* (%)	33 (51.56)	69 (71.13)	0.017	56 (31.28)	9 (56.25)	0.712
Poor prognosis (mRS 3–5) 3 month after ICH, *n* (%)	31 (48.44)	28 (28.87)	0.035	123 (68.72)	7 (43.75)	0.042
Mortality (mRS 6) within 3 months after ICH, *n* (%)	6 (9.38)	0	NA	35	0	NA
Brain MRI performed, *n* (%)	64 (100)	70 (72.16)	NA	109 (60.89)	9 (56.25)	NA

Values are presented as the number (%) or mean ± standard deviation. *p* values < 0.05 were considered statistically significant based on the Chi square test and Student’s *t*-test. ICH, intracerebral hemorrhage; cSVD, cerebral small vessel disease; mRS, modified Rankin scale; SVD+, SVD present; and SVD−, SVD absent.

**Table 2:** Neuroimaging classification in patients with ICH (*n* = 356).

	Age < 65 Years (*n* = 161)			Age ≥ 65 Years (*n* = 195)		
	cSVD+ (*n* = 64)	cSVD− (*n* = 97)	*p*-Value	cSVD+ (*n* = 179)	cSVD− (*n* = 16)	*p*-Value
Age (year)	54.67 ± 8.44	48.68 ± 10.82	<0.001	78.90 ± 7.59	67.1 ± 3.9	<0.001
Male, *n* (%)	45 (70.31)	56 (57.73)	0.03	104 (58.10)	7 (43.75)	0.051
ICH score (0–4)	1.84 ± 1.50	1.54 ± 1.38	0.041	2.20 ± 1.31	1.5 ± 1.35	0.007
**ICH etiology**						
Hypertension, *n* (%)	56 (87.5)	72 (74.23)	0.02	116 (64.8)	9 (56.25)	0.078
Cerebral amyloid angiopathy, *n* (%)	1 (1.56)	1 (1.03)	0.872	49 (27.37)	4 (25.0)	<0.001
Arteriovenous malformation, *n* (%)	0 (0.0)	11 (11.34)	0.516	0 (0.0)	0 (0.0)	0.947
Moyamoya disease, *n* (%)	6 (9.36)	4 (4.12)	0.045	3 (1.68)	0 (0.0)	0.01
Tumor-related hemorrhage, *n* (%)	1 (1.56)	4 (4.12)	0.217	6 (3.35)	1 (6.25)	0.263
Unknown cause, *n* (%)	0 (0.0%)	5 (5.15)	0.889	5 (2.79)	2 (12.5)	0.747
**ICH location**						
Basal ganglia, *n* (%)	26 (46.63)	46 (47.42)	0.641	49 9 (27.37)	4 (25.0)	0.562
Thalamus, *n* (%)	14 (21.88)	10 (10.31)	0.032	38 (21.23)	2 (12.5)	0.153
Cerebral lobe, *n* (%)	11 (17.19)	23 (23.71)	0.525	64 (35.75)	5 (31.25)	0.082
Pons and brainstem, *n* (%)	9 (14.06)	8 (8.25)	0.094	12 (6.70)	0 (0.0)	<0.001
Cerebellum, *n* (%)	4 (6.25)	10	<0.001	16 (8.94)	5 (31.25)	0.074
**cSVD classification**						
Presence of white matter hyperintensity, *n* (%)	49 (76.56)	NA		152 (84.92)	NA	
Presence of lacunes, *n* (%)	23 (35.94)			91 (50.84)		
Presence of microbleeds, *n* (%)	33 (51.56)			76 (42.46)		
Presence of enlarged perivascular spaces, *n* (%)	1 (1.56)			19 (10.61)		
SVD score (0–6)	1.73 ± 0.67			2.18 ± 0.90		

Values are presented as the number (%) or mean ± standard deviation. *p* values < 0.05 were considered statistically significant based on the Chi square test and Student’s *t*-test. ICH, intracerebral hemorrhage; cSVD, cerebral small vessel disease.

**Table 3:** Comparison of functional improvement in ICH patients with and without SVD stratified by age.

	Age < 65 Years (*n* = 161)			Age ≥ 65 Years (*n* = 195)		
Function	cSVD+ (*n* = 64)	cSVD− (*n* = 97)	*p*-Value	cSVD+ (*n* = 179)	cSVD− (*n* = 16)	*p*-Value
**Initial Evaluation**						
mRS	3.78 ± 1.20	3.41 ± 0.92	0.018	4.13 ± 0.92	3.8 ± 0.92	<0.001
MBI (daily activity)	26.98 ± 27.33	20.5 ± 24.27	0.006	16.25 ± 20.60	20.5 ± 24.27	<0.001
BBS (balance and gait)	14.17 ± 18.65	10.7 ± 15.37	0.032	7.07 ± 12.84	10.7 ± 15.37	<0.001
FAC (gait)	1.20 ± 1.62	0.6 ± 0.97	0.187	0.56 ± 1.01	0.6 ± 0.97	0.754
MFT (hand function)	13.25 ± 10.92	9.4 ± 9.71	0.019	10.41 ± 9.40	9.4 ± 9.71	0.912
Swallowing function (non-oral diet/limited diet/normal diet)	16/13/35 (25.0/20.31/ 54.69%)	2/35/60 (2.06/36.08/ 61.86%)	0.415	117/43/19 (65.36/24.02/10.61%)	2/9/5 (12.5/56.25/ 31.25%)	0.076
**Follow-Up Evaluation at three months after ICH**						
mRS	2.92 ± 1.75	2.13 ± 1.60	0.013	3.71 ± 1.62	2.8 ± 0.82	<0.001
MBI (daily activity)	47.20 ± 34.98	60.62 ± 33.29	0.019	28.5 ± 28.67	44.21 ± 29.42	<0.001
BBS (balance and gait)	26.94 ± 20.95	33.33 ± 21.53	0.072	13.80 ± 17.53	22.62 ± 20.19	<0.001
FAC (gait)	2.38 ± 1.84	2.95 ± 1.70	0.05	1.30 ± 1.43	1.92 ± 1.20	<0.001
MFT (hand function)	18.86 ± 12.51	22.82 ± 11.07	0.043	14.64 ± 11.34	22.51 ± 10.22	<0.001
Swallowing function (non-oral diet/limited diet/normal diet)	8/11/45 (12.5/17.19/ 70.31%)	0/8/89 (0.0/8.25/91.75%)	0.002	98/50/31 (54.75/27.93/17.32%)	0/5/11 (0.0/31.25/68.75%)	0.021
Hospital admission status at 3 months after ICH, *n* (%)	12 (18.75%)	3 (3.09%)	0.179	138 (77.19%)	8 (50.0%)	0.523

Values are the mean ± standard deviation or number (%). *p* values < 0.05 were considered statistically significant based on Student’s *t*-test or Chi square test. mRS: modified Rankin scale, MBI: modified Barthel index, BBS: Berg balance scale, FAC: functional ambulatory category, MFT: manual function test, ICH: intracerebral hemorrhage.

**Table 4:** Multivariable logistic regression analysis for predictors of poor prognosis stratified by age.

	Age < 65 Years (*n* = 161)			Age ≥ 65 Years (*n* = 195)		
Variable	Odds Ratio	95% CI	*p*-Value	OR	95% CI	*p*-Value
**cSVD present**	3.82	1.23–8.76	0.004	7.44	2.40–15.35	<0.001
**ICH score**	3.95	2.51–6.21	<0.001	3.29	2.36–4.89	<0.001
**Age (per year)**	1.04	1.01–1.09	0.02	0.96	0.91–1.01	0.117
**Chronic kidney disease**	4.21	1.35–7.61	0.02	5.48	1.43–13.72	0.03
**Hypertension**	2.95	1.05–4.26	0.03	2.17	1.74–3.49	<0.001
**Diabetes**	3.16	1.12–6.94	0.02	1.96	0.97–10.91	0.152
**Heart failure**	0.74	0.61–4.96	0.527	1.35	0.82–5.34	0.415

CI: confidence interval, cSVD: cerebral small vessel disease, ICH: intracerebral hemorrhage, *p* values < 0.05 were considered statistically significant based on the multivariable logistic regression.

## Data Availability

The data from this study are available from the corresponding author on reasonable request.

## Abbreviations

The following abbreviations are used in this manuscript:

ADL	activities of daily living
BBS	Berg balance scale
cSVD	cerebral small vessel disease
FAC	functional ambulatory category
MBI	modified Barthel index
MFT	manual function test
mRS	modified Rankin scale
ICH	intracerebral hemorrhage
WMHs	white matter hyperintensities
